# Type I Interferons as Regulators of Human Antigen Presenting Cell Functions

**DOI:** 10.3390/toxins6061696

**Published:** 2014-05-26

**Authors:** Sandra Gessani, Lucia Conti, Manuela Del Cornò, Filippo Belardelli

**Affiliations:** Department of Hematology, Oncology and Molecular Medicine, Istituto Superiore di Sanità, Viale Regina Elena 299, Rome 00161, Italy; E-Mails: lucia.conti@iss.it (L.C.); manuela.delcorno@iss.it (M.D.C.); filippo.belardelli@iss.it (F.B.)

**Keywords:** type I interferon, dendritic cell, cell differentiation/activation, antigen uptake/processing/presentation, T cell response, transcriptional profile, microRNA

## Abstract

Type I interferons (IFNs) are pleiotropic cytokines, initially described for their antiviral activity. These cytokines exhibit a long record of clinical use in patients with some types of cancer, viral infections and chronic inflammatory diseases. It is now well established that IFN action mostly relies on their ability to modulate host innate and adaptive immune responses. Work in recent years has begun to elucidate the mechanisms by which type I IFNs modify the immune response, and this is now recognized to be due to effects on multiple cell types, including monocytes, dendritic cells (DCs), NK cells, T and B lymphocytes. An ensemble of results from both animal models and *in vitro* studies emphasized the key role of type I IFNs in the development and function of DCs, suggesting the existence of a natural alliance between these cytokines and DCs in linking innate to adaptive immunity. The identification of IFN signatures in DCs and their dysregulation under pathological conditions will therefore be pivotal to decipher the complexity of this DC-IFN interaction and to better exploit the therapeutic potential of these cells.

## 1. Introduction

Interferons (IFNs) were first discovered as an important anti-viral factor that interferes with viral replication in mammalian cells. Three classes of IFNs (Type I, II, and III) have been identified and categorized on the basis of their structural homology and the specific receptor they associate with [1]. The type I IFN family is very diverse and includes numerous IFN-α variants (13 in human and 14 in mouse), a single IFN-β member, and the lesser known IFN-ε, -k, -ω, and -δ [2]. Despite this diversity, all type I IFNs bind exclusively to the IFN-α receptor (IFNAR) [3]. In the history of cytokine research, IFN-α has represented a “moving target” since a variety of different and even opposite biological effects have been described over the years for these cytokines, including antitumor activity and modulation of multiple immune and non-immune cell functions [4,5,6]. The importance of the effects of type I IFNs on cells of the immune system has been neglected for a long time [7]. However, work in recent years has begun to elucidate the mechanisms underlying type I IFN ability to influence the adaptive immune response, and this is now recognized to be due to effects on multiple cell types [8,9,10,11]. Early studies had reported the type I IFN-mediated enhancement of macrophage functions as well as of NK cell activity [11,12]. Subsequent studies outlined the importance of these cytokines in the modulation of T cell functions, including the polarization of T helper (Th) cells toward the Th1 type, and the generation/activation of cytotoxic T lymphocytes (CTL) [8,13]. Over the last years, it has become evident that many of the effects of type I IFNs on adaptive immunity are mediated by effects of these cytokines on macrophages and dendritic cells (DCs).

DCs are a heterogeneous, sparsely distributed population of bone marrow-derived immune cells, that play an essential role in the induction and control of immunity [14]. Since their initial description by Steinman and Cohn [15], the existence of many distinct DC subtypes has now become evident [16,17]. Tissue microenvironments appear to have a major impact on the development and function of resident DCs, determining which type of T cell response is induced [18,19,20]. As high amounts of IFN can be physiologically produced in response to infectious agents and inflammatory stimuli, these cytokines may be among the factors signalling danger to circulating monocytes, thus enabling them to rapidly differentiate into DCs. Some groups, including ours, have shown that type I IFNs promote the differentiation of monocytes into DCs, leading to the generation of DC subsets endowed with distinct and potent functional activities [21,22,23,24]. This points to the existence of a “natural alliance” between type I IFNs and monocytes/DCs, which might be instrumental for the prompt generation of a protective immune response against pathogens as well as against cancer cells. In particular, the exposure of monocytes to type I IFNs can represent an early mechanism driving DC activation in response to virus infection and possibly to other invading pathogens or tumors.

In this review, we provide an overview of the existing knowledge that antigen presenting cells (APCs) represent a direct target of type I IFNs. We will focus on human DCs and discuss the effects of type I IFNs on the biology of these cells, including differentiation, functional activation, and transcriptional signature.

## 2. Type I IFNs in DC Development and Activation

**Figure 1:**
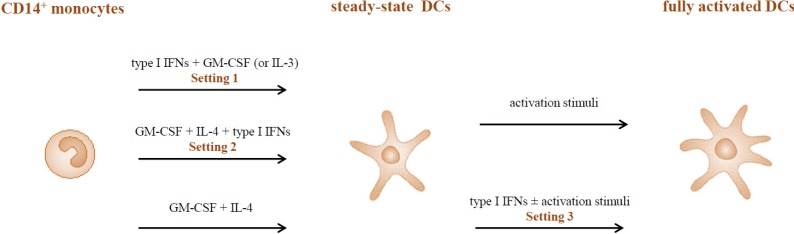
Type I interferons (IFN)-antigen presenting cells (APC) interaction in different experimental settings. A schematic representation of the different experimental settings usedto test the effects of type I IFNs on the generation/activation of human dendritic cells (DCs).

The role of type I IFNs in the development of DCs as well as in the regulation of their functional activity has been investigated in a large number of studies. However, differences in the experimental setting, timing of IFN addition and culture conditions, render somehow difficult a direct comparison of results, yielding in some cases apparently contradictory data. As shown in Figure 1, different experimental settings have been used to test the effect of type I IFNs on the generation/activation of human DCs. Many studies investigated the effect of these cytokines, added together with either GM-CSF or IL-3 to freshly isolated monocytes (Setting 1). The phenotype and function of IFN-generated DCs (IFN-DCs) were characterized at the steady-state as well as upon further stimulation by activation/maturation factors. Conversely, after the report by Luft and co-workers [24] that type I IFNs accelerate the differentiation of CD34^+^ progenitors to DCs when added together with GM-CSF, IL-4 and TNF-α, subsequent studies investigated the influence of IFNs in the differentiation of DCs with GM-CSF and IL-4, referred to as IL-4-DCs (Setting 2). As above, DCs generated under these experimental conditions were analyzed as such or after further activation. Finally, type I IFNs were also tested for their ability to affect the maturation of IL-4-DCs, either alone or in combination with other activation stimuli (Setting 3).

In this section, we will review evidence on how type I IFNs regulate the phenotype, migratory properties and soluble mediator profile of DCs obtained with the 3 different experimental settings.

### 2.1. Type I IFNs Promote the in Vitro Generation of DCs (Setting 1)

Since the report by Paquette and colleagues [22], the majority of studies, including ours, confirmed and extended the finding that type I IFNs, mostly IFN-α, in combination with GM-CSF or IL-3, promote the differentiation of DCs from both monocytes and CD34^+^ hematopoietic progenitors, leading to a deep characterization of their immunophenotype and functional features. Table 1 lists the main phenotypic and functional properties of IFN-DCs (Setting 1). Exposure of monocytes to GM-CSF plus type I IFNs leads, within 3 days, to loss of plastic adherence associated with cellular aggregation in large clusters, and appearance of typical DC morphology [21,22,25]. These cells express major histocompatibility complex (MHC) molecules class I and II, co-stimulatory markers (CD25, CD40, CD80 and CD86), adhesion molecules (CD54, CD58) [21,22,25,26,27] and cellular factors involved in antigen uptake and processing, CD8^+^ T cell cross-priming, and in priming of CD4^+^ T lymphocytes [21,22,25,26,27,28]. Conversely, low levels of expression of CD14 are retained during the differentiation process [21,22,29]. The IFN-induced differentiation is irreversible and, in contrast to that driven by GM-CSF and IL-4, persists upon removal of the cytokines [21,25]. In addition to IFN-α, the combination of GM-CSF and IFN-β turned out to be very efficient in inducing monocyte differentiation into DCs endowed with phenotypic features very similar to those observed in the presence of IFN-α [30,31,32]. In some studies, GM-CSF was replaced by IL-3, yielding DCs with mostly, but not completely, overlapping features [29,31,33].

**Table 1:** Phenotypic and functional properties of IFN-DCs.

Properties	Partially activated	Fully activated	Ref.
Co-stimulatory molecules	CD25, CD40, CD80, CD86	↑	[21,22,25,26,27,28,29]
MHC	Class I (A,B,C), class II (DR)	↑	
Adhesion molecules	CD44, CD54, CD58, LFA-1	↑	
Macrophage markers	CD14	N.D.	
pDC markers	CD123, BDCA1, BDCA4	N.D.	[21,22,25,26,27,29,30,33,34,47]
NK cell markers	TRAIL CD56, granzyme B and M, defensin-α1	↑	[21,26,34]
Activation markers	CD83	↑	[21,25,28,35,36]
TLR	1,2,3,4,5,6	↓	[27,36]
	7,8	↑↓	
Chemokine receptors	CXCR4, CCR2, CCR5, CCR7	N.D.	[28,31,37,38,39]
Cytokines	TNF-α, IL-1β, IL-6, IL-8, IL-10, IL-12 p40, IL-15, IL-18, intracellular IFN-γ	↑ IL-12 p70, IL-1β, TNF-α, IL-6, IL-10, IL-15, IL-18, IL-23, IL-27, IL-1RA, type I IFNs	[21,25,27,28,31,33,34,35,36,43]
Chemokines	CXCL10, CCL2, CCL8, CCL19	↑ CCL2, CCL3, CCL4	[35,37]
Migratory activity	CCL3, CCL4, CCL5, CCL19, CCL21	N.D.	[37,38,39]
Antigen uptake	LOX-1	↓ LOX-1	[27,28,40,41]
	DC-SIGN, MR, CLEC7A/Dectin1		
Antigen processing/presentation	CD208/DC-LAMP, TAP-1/2, tapasin, PA28α, PA28β, LMP-2, LMP-7, MECL1 MHC I	↑ MHC I	[21,27,40,42,43,44]
T cell polarization	Th0, Th1, Th2, Tr	↑ Th0, Th1, Th17	[21,25,30,33,42,45,46,47]

Note: A summary of the phenotypic and functional features of DCs generated in the presence of type I IFN subtypes at the steady-state and upon induction of full activation (Setting 1); N.D. = not determined.

IFN-DCs exhibit a combined phenotype as they display myeloid and plasmacytoid DC (pDC) features associated with NK cell characteristics. In addition to the expression of CD123 [21,25,26,27,29,30,33], BDCA4 and low levels of CD209/DC-SIGN [25,27], these cells also express CD56 [26,34] as well as cytotoxic effector molecules, like the granzymes B and M, TRAIL and defensin-α1, which are important components of the cytotoxic arsenal of NK cells [21,26].

IFN-DCs exhibit an intrinsic attitude to undergo apoptosis when cultured for more than 3 days. Conversely, IL-4-DCs do not spontaneously undergo apoptosis unless stimulated with LPS. This different behavior relies on the constitutive expression of the apoptosis-inducing molecule TRAIL in IFN-DCs, whereas in IL-4-DCs the expression of this molecule is induced only following LPS addition [21]. As a consequence of TRAIL expression, IFN-DCs are capable of specifically killing TRAIL-sensitive tumor cells [21].

At the steady-state, IFN-DCs exhibit the phenotype of partially mature DCs, as evidenced by a consistent expression of low levels of CD83 and CD25 [21,25,27,28,35,36], and the presence of CD44^+^, highly polarized, thin and long dendrites [37]. IFN-DCs spontaneously produce IL-15 [21], IL-18 [27] and little amounts of IL-10 [45]. Consistently with their NK cell features, intracytoplasmatic cytokine staining revealed that the majority of IFN-DCs, independently of their CD56 expression, are IFN-γ positive as well [34]. IFN-DCs generated in the presence of IL-3 and IFN-β spontaneously produce TNF-α, IL-6, IL-8 and little amounts of IL-12p40 [33].

IFN-DCs express numerous proinflammatory and homeostatic C-C and C-X-C motif chemokines [21,37,48] which are involved in the recruitment and activation of a variety of immune cells and promote angiogenesis. A prevalent expression of Th1 recruiting chemokines, including CCL18 and CXCL10, was found in IFN-DCs with respect to classically generated cells [37]. With respect to the migratory behavior, IFN-DCs not only express very high levels of CCR5, but also exhibit an enhanced migratory response to its ligands, the inflammatory chemokines CCL3, CCL4 and CCL5 [28,31,37,38,39]. Furthermore, IFN-DCs express CXCR4 and CCR2 [28,31]. Consistently with their partially mature phenotype, a remarkable fraction of IFN-DCs proved to express CCR7 [31,37,38,39] and to show a migratory response to CCL19 [37] and CCL21 [39], chemokines regulating DC trafficking to secondary lymphoid organs. Type I IFNs also change the adhesion and migratory properties of DCs in their interplay with lymphatic endothelium. Differentiation of DCs in the presence of type I IFNs augments LFA-1 expression levels and promotes the appearance of an epitope on the integrin chain that indicates an active molecular conformation [49]. Interestingly, the enhanced migratory response to CCL3 observed in DCs differentiated in the presence of IFN-α2b and IFN-α5 indeed involved LFA-1 [39].

Lastly, IFN-DCs also express a large number of TLRs including TLR1, 2, 3, 4, 5, 6, and 8. Notably, in marked contrast to IL-4-DCs, IFN-DCs also express high levels of TLR7 [27,36], which is classically found in pDCs, whereas they do not express TLR9.

In spite of their “partially” mature phenotype, IFN-DCs proved to be fully susceptible to undergo activation/terminal differentiation after stimulation with TLR (*i.e.*, LPS, polyI-C, ssRNA) or CD40 ligands, as revealed by the enhanced expression of accessory molecules as well as by a massive CD83 induction [21,25,36]. Upon TLR or CD40 triggering, IFN-DCs release IL-12p70, IL-23, IL-27, IL-1β, IL-6 and TNF-α and increase the expression of IL-10, IL-15 and IL-18 [21,27,28,36]. Interestingly, it has been recognized that many of the stimuli promoting DC maturation, including TLR ligands, monocyte conditioned medium, HSV or imiquimod, also induce the production of large amounts of type I IFNs [25,27,31,50]. Although these observations suggested that type I IFN induction in IFN-DCs might contribute to their Th1 promoting capacity, only a partial reduction of IFN-γ production in primed T lymphocytes is observed following its neutralization [27]. Likewise, blocking LPS-induced endogenous type I IFNs does not interfere with the acquisition of a fully mature phenotype, nor has a significant effect on the allostimulatory properties of LPS-stimulated IFN-DCs [50].

### 2.2. Adjuvant versus Inhibitory Effects of Type I IFNs on the Differentiation of IL-4-DCs (Setting 2)

A number of studies investigated the influence of type I IFNs on the GM-CSF/IL-4 driven differentiation of monocytes to DCs (Setting 2). Typically, IFNs were added at initiation of culture, either alone or combined to other agents (TLR ligands, TNF-α). Early reports demonstrated that the addition of type I IFNs to monocyte cultures containing optimal concentrations of IL-4 and GM-CSF markedly increases CD86 and HLA-DR expression levels above those present in DCs grown without IFNs [22]. Likewise, addition of IFN-α at the time of culture initiation greatly increases both the number of mature DCs generated and their rate of appearance. Indeed, by 3 days of culture, many large floating aggregates containing mature CD83^+^, CD1a^low^ DCs are present, while much fewer aggregates of mature DCs are found without IFN-α. This effect relies on the observed synergy between IFN-α and IL-4 [23].

Subsequent studies investigated the effects of the combined action of type I IFNs and microbial products (LPS or LTA) on DC differentiation and survival. This experimental setting revealed a sensitivity of DCs to activation induced cell death if generated in the presence of type I IFNs [51].

In contrast with the above described studies, it was found that type I IFN addition to GM-CSF/IL-4 monocyte cultures soon after seeding reduces the survival and alters the morphology and differentiation of DCs. Upon maturation induction with TNF-α, IFN-β-generated immature DCs exhibit a reduced expression of CD1a, CD40, CD54 and CD80 as well as lower allostimulatory capacity and production of IL-12 [52]. Conversely, Bartholomè and colleagues reported that, although type I IFNs do not inhibit but rather stimulate MHC II, CD80 and CD86 expression, and do not alter DC morphology when added at initiation of culture, a marked and selective reduction in the CD40 ligand-stimulated IL-12 production was observed [53]. Likewise, Hussien and co-workers reported that type I IFN addition rapidly promotes the development of activated DCs as reflected by up-regulation of MHC II and CD86. However, it also reduces IL-12 spontaneous secretion while increasing that of IL-10 [54]. Likewise, Dauer and colleagues reported that incubating GM-CSF/IL-4 cultured monocytes with IFNs does not affect phenotypic maturation of DCs but reduces IL-12 production upon activation [55].

### 2.3. Type I IFNs as Activation Stimuli for Immature DCs (Setting 3)

The capacity of type I IFNs to modulate DC activation/maturation has been investigated in several studies. In reviewing them, special attention should be given to the type of DCs generated (*i.e.*, cytokine cocktail) as well as to the concomitant presence of other activation stimuli (Setting 3). A list of the main observations achieved under these experimental conditions is shown in Table 2. Early evidence of IFN-induced DC maturation was provided by Luft and colleagues who reported that type I IFNs accelerate the maturation of immature DCs obtained from CD34^+^ hematopoietic progenitors. They also showed that low levels of endogenous IFN are released in the culture medium of spontaneously maturing DCs, suggesting a role of these cytokines in the maturation process [24]. Using a rather different approach for investigating the effects of IFNs on circulating blood DCs, Ito and co-workers also reported that IFN-α markedly enhances the maturation of CD11c^+^ DCs [56]. However, type I IFNs turned out to be poor inducers of maturation when added to IL-4-DCs as compared to other stimuli. These cytokines up-regulate MHC and co-stimulatory molecules but not CD83, partially reduce antigen-uptake function, increase the levels of IL-12p35 mRNA and prolong surface expression of peptide-MHC I complexes for presentation to CD8^+^ T cells, but do not induce CCL21-mediated migration [57]. Conversely, a clear-cut synergy was observed when type I IFNs were added together with other maturation stimuli, both microbial and T cell derived, as well as other cytokines. Specifically, type I IFNs act synergistically with TNF-α to induce DC maturation [23]. Likewise, addition of type I IFNs to the standard DC maturation cocktail up-modulates CD83 and CD38 and improves the induction of autologous T cell responses, despite a few other changes in DC phenotype and cytokine secretion [58]. Luft and colleagues reported that type I IFNs strongly enhance CD40L-mediated DC maturation by shifting the IL-12p70/p40 ratio toward the bioactive p70 form [57]. Interestingly, IFN-exposed IL-4-DCs strongly promote MHC I-restricted responses and produce high amounts of CXCL9 and CXCL10, which may exert a chemotactic effect on CD8^+^ effector T cells [48,59]. Addition of IFN-α to TLR stimulation further enhances the expression of CD83, CD38, and CCR7 as well as the ability of maturing DCs to produce IL-12p70 and to migrate in response to CCL21, used as a ligand of CCR7 [60]. Lastly, Lehner and colleagues showed a strong synergistic effect between type I IFNs and products from gram-negative and gram-positive bacteria for the induction of apoptosis. This effect occurs if both stimuli are present together during the whole culture, but also when the bacterial stimulus is added later to immature DCs generated in the presence of type I IFNs [51]. Some apparently contradictory results can be at least in part explained by the “timing” of IFN addition.

**Table 2:** Effects of type I IFNs on DC activation/maturation.

Observation	Culture conditions	Stimulus	Ref.
↑ phenotypic activation (CD80, CD86, MHC I and II)	CD34^+^ progenitors derived DCs	IFN-α2, α8, β	[24]
↓ CD1a, CD11c			
↑ phenotypic activation (CD86, MHC II)	IL-4-DCs	IFN-α	[22]
↑ phenotypic (CD40, CD86, CD83, MHC II) and functional activation	IL-4-DCs + TNF-α	IFN-α2b	[23]
↓ CD1a			
↑ phenotypic (CD80, CD86, MHC I and II) and functional (CD8 priming) activation = CD83 and CCR7 expression	IL-4-DCs	IFN-α2a	[48,59]
↑ CXCL19 and CXCL10			
↑ CD38, CD83low	IL-4-DCs	IFN-α	[58]
↑ CD83, CD38low		IFN-α + TNF-α/IL-1β/IL-6/PGE_2_	
↑ CD38, CD83, CCR7	IL-4-DCs	IFN-α + TLR ligands	[60]
↑ IL-12 secretion			
↑ apoptosis	IL-4-DCs	IFN-α + microbial stimulation	[51]
↓ phagocytosis	IL-4-DCs	IFN-α2a + CD40L or IFN-α2a + PGE_2_ + TNF-α	[57]
↑ CCR7 expression			
↑ IL-12 secretion			
↑ T cell IFN-γ production			
↑ MHC II expression	GM-CSF cultured	IFN-α2a, IFN-β	[61]
↑ MHC II-restricted presentation	myeloid DCs		
↑ antigen uptake and cross-presentation	IL-4-DCs	IFN-α	[22]
↑ cross-presentation	IL-4-DCs + TNF-α/PGE_2_/antigen	IFN-α, IFN-αβ	[62]
↓ cross-presentation	IL-4-DCs	IFN-α + TNF-α + PGE_2_ + Ag	[62]

Note: A summary of the effects of type I IFN subtypes, either administered alone or in combination with other stimuli, on the activation of IL-4-DCs (Setting 3).

In this regard, by using an in vitro model of DC-dependent, human naïve Th cell differentiation, it has been demonstrated that while maturation in the presence of IFN-β generates DCs that strongly promote polarization toward IFN-γ-secreting T cells, exposure to IFN-β during mature DC-mediated primary stimulation of naïve Th cells significantly inhibits Th1 cell polarization. The mechanism by which IFN-β mediates these contrasting effects is based on differential regulation of IL-12 family cytokine and IL-18 secretion [63]. Likewise, it has been reported that IFN-β has both immunostimulatory and immunosuppressive effects on DCs depending on the stage of maturation. When IFN-β is added together with GM-CSF and IL-4 to monocytes at the beginning of culture, apoptosis is induced and thereby the development of DCs is inhibited. In contrast, stimulation of immature DCs at day 7 of culture with IFN-β results in a concentration-dependent up-regulation particularly of CD86, but also of CD80 and MHC II. Moreover, IFN-β enhances the capacity of DCs to stimulate autologous T cells to secrete IL-13, IL-10 and IL-5 [64].

## 3. Type I IFNs in Antigen Uptake/Processing and T Cell Response Generation

The highly regulated expression of costimulatory molecules and cytokines in APCs reflects the critical importance of these cells in determining the type of immune response induced and in shaping adaptive immunity. Two generally distinct pathways are used by MHC I and II molecules for the presentation of peptide antigens to CD8^+^ and CD4^+^ T cells, respectively [65]. The MHC I-restricted pathway is active in almost all cell types and allows the presentation of peptides derived from endogenously synthesized proteins. Conversely, the MHC II pathway is constitutively active only in “professional” APCs and provides a way for CD4^+^ T cells to respond to exogenous antigens internalized through different mechanisms (e.g., phagocytosis, macropinocytosis, receptor-mediated endocytosis and others). In the recent years, several studies highlighted DC and macrophage ability to present exogenous antigens, internalized through the endocytic pathway, to CD8^+^ T lymphocytes through the process of cross-presentation. The mechanisms responsible for such cross-priming turned out to be of critical importance for initiating CD8^+^ T cell responses against pathogen- and tumor cell-derived antigens that would not otherwise gain access to the MHC I pathway [65].

A major component of the antiviral and antitumor properties of type I IFNs is the regulation of genes and pathways involved in the processing and presentation of antigens in DCs, which confers these cells the capacity to rapidly and efficiently stimulate antigen-specific CD4^+^ and CD8^+^ T cell responses. Several studies in animal models as well as in *in vitro* cultured DCs have highlighted the crucial role of type I IFNs in the regulation of both MHC I- and MHC II-restricted antigen presentation pathways, as well as their special attitude to stimulate cross-presentation. As reviewed in this section, the enhancing effect of type I IFNs is achieved through the control of the expression and activation status of a number of molecules involved in the recognition, processing, transport and final presentation of antigens to T lymphocytes.

### 3.1. Antigen Recognition and Internalization

The type of antigen and the route by which it is internalized in APCs finally determine the fate of T cell response induced. While antigens internalized through scavenger receptors or pinocytosis are targeted to lysosomes and presented on MHC II to CD4^+^ T cells, those captured through mannose receptor (MR)-mediated endocytosis or macropinocytosis are mainly targeted to early endosomes, resulting in cross-presentation to CD8^+^ T cells [65]. The effects of type I IFNs on the initial steps of antigen recognition and internalization have been studied and a major finding is represented by the selective expression of the scavenger receptor oxidized low-density lipoprotein receptor 1 (LOX-1) in IFN-DCs [40] (Table 1). IFN-induced LOX-1 mediates the engulfment of apoptotic cells and strongly contributes to the peculiar ability of IFN-DCs to present apoptotic tumor cell-derived antigens in both MHC I- and II-restricted fashion [40]. In a recent paper by Garcin and co-workers [32], it has been shown that IFN-β-generated DCs are less potent than those generated with IFN-α or IFN-ω in the phagocytosis of both apoptotic and necrotic cells. This correlates with a reduced expression of genes for phagocytosis receptors (FCGR, MARCO, CLEC5A) in IFN-β-generated DCs, highlighting a different capacity of the type I IFN subtypes to regulate dead cell recognition and internalization.

Concerning the uptake of protein antigens for cross-presentation, no evident difference in MR-mediated endocytosis was observed between IFN-DCs and IL-4-DCs, thus excluding any role of these cytokines in regulating protein antigen uptake/internalization, despite their enhancing effect on cross-presentation [22,25,42]. Subsequently, we reported that the expression of MR, as well as of the β-glucan receptor CLEC7A/Dectin-1, is comparable in the two DC subtypes [41]. Furthermore, in contrast to other maturation stimuli, IFN-β does not affect the expression of these receptors when used as activation stimulus for IL-4-DCs (Setting 3) [41]. It is worth mentioning that IFN-α and -β strongly down-regulate MR and Dectin-1 expression in human monocyte-derived macrophages and this results in impaired protein antigen endocytosis and pathogen phagocytosis [41]. Consistently, infection of alveolar macrophages with influenza A virus has been associated with a marked down-regulation of MR and Dectin-1 mRNA expression as well as of zymosan phagocytosis, concomitantly with a strong type I IFN response [66]. Furthermore, recent data obtained with IFN-α-stimulated bone marrow (BM)-derived mouse DCs show a reduced MR-mediated antigen uptake upon cytokine addition [67]. These different results outline an opposite regulation of antigen uptake/endocytosis by type I IFNs in human and mouse, depending on the type of APC.

### 3.2. Antigen Processing for Cross-Presentation and CD8^+^ T Cell Priming

A number of studies have reported the ability of type I IFNs to promote CD8^+^ T cell cross-priming against viral and tumor antigens through the stimulation of DCs [68,69]. The enhancing effect results from a pleiotropic activity of these cytokines on different steps of the antigen presentation pathway, spanning from enhancement of endosomal antigen processing and transport to up-regulation of MHC and co-stimulatory molecules, and increased DC viability (Table 1 and Table 2).

Pivotal studies demonstrating the peculiar enhancing effect of type I IFNs on cross-presentation were performed in mouse DCs, either upon *in vivo* IFN induction or *in vitro* stimulation. In particular, following the initial observation that virus infection enables mouse splenic DCs for CD8^+^ T cell cross-priming through IFN-α/β production [68], type I IFNs were subsequently shown to mediate CpG DNA-, dsRNA- and LPS-induced enhancement of antigen cross-presentation by increasing MHC I mRNA transcription and stability [70,71,72]. *In vivo* studies in IFN-β or IFNAR deficient mice confirmed the crucial role of endogenous type I IFNs in regulating MHC I-peptide complex formation and Hsp70 expression [73].

The ensemble of results obtained in mouse models have been instrumental for subsequent studies performed with IFN-differentiated/activated human DCs. By comparing DCs generated in the presence of IFN-α (Setting 1) with IL-4-DCs, our group initially identified the peculiar capacity of IFN-DCs to induce *in vivo* cross-priming of CD8^+^ T cells against exogenous viral antigens [42,46]. These cells express higher levels of MHC I and efficiently stimulate CD8^+^ T cell activation at lower antigen concentration as compared to IL-4-DCs, even following CD40 stimulation of these latter. As highlighted above, neither MR-mediated endocytosis nor OVA processing are differently regulated in the two DC subtypes [42].

IFN-DCs have subsequently been reported to easily recognize apoptotic tumor cells and to efficiently cross-present tumor-derived antigens to CD8^+^ T cells, thus leading to an increased cross-priming also against tumor-associated antigens [40]. In this contest, together with the up-regulation of molecules involved in antigen transport and presentation (MHC I, TAP-1, LMP-2, CD208), a high and selective surface expression of the Hsp70-binding receptor LOX-1 was found in IFN-DCs as compared to reference IL-4-DCs [40]. LOX-1 was found to be essential for the uptake of apoptotic cells by IFN-DCs and for the subsequent presentation of tumor-derived antigens to both CD8^+^ and CD4^+^ T cells. These findings point to IFN-α as a signal that instructs DCs to activate intracellular pathways for the simultaneous MHC I- and II–restricted presentation. Parallel studies identified type I IFNs as strong promoters of *in vivo* CD8^+^ T cell cross-priming against tumor apoptotic cell-derived antigens in the mouse system [74].

Concerning the mechanisms underlying the special attitude of IFN-DCs in performing cross-presentation, our group has further shown that IFN-α boosts cross-presentation of viral and tumor-associated antigens by DCs via the modulation of proteasome activity. Specifically, steady-state IFN-DCs exhibit comparable levels of the constitutive α2 proteasome subunit, but enhanced expression of the PA28α and PA28β regulator subunits and of the inducible catalytic subunits LMP2, LMP7 and MECL1, with respect to immature IL-4-DCs [43]. The levels of expression/activation of these molecules are comparable to those achieved in IL-4 DCs upon LPS stimulation [43]. Moreover, superior levels of TAP1, TAP2 and tapasin, together with higher proteolitic activity, have been found in IFN-DCs [43] (Table 1). Previous studies had shown that IFN-DCs preferentially express inducible immunoproteasome subunits, in contrast to IL-4-DCs that also express constitutive subunits [44]. More recently, the cross-presentation process has been further investigated in IFN-DCs by studying the intracellular sorting of both soluble protein antigen (e.g., OVA) and nonstructural-3 protein of Hepatitis C virus (HCV) [75]. These studies have clearly demonstrated that, independently of the route and mechanism of antigen delivery, IFN-DCs are extraordinarily competent in preserving internalized proteins from early degradation and in routing antigens toward the MHC I processing pathway, thus allowing long-lasting cross-priming capacity.

Type I IFNs regulate the cross-presentation of exogenous antigens even when used as activation stimuli for IL-4-DCs (Setting 3), and exert positive or negative effects depending on the maturation stage of DCs. In particular, Longman and co-workers [62] demonstrated that exposure of immature DCs to IFN-α/β concomitantly with antigenic apoptotic cells and maturation stimuli (TNF-α/PGE2) impairs their ability to cross-prime CD8^+^ T cells. Conversely IFN-exposed mature DCs, immediately before T cell engagement, exhibit an enhanced cross-presentation activity. These opposite effects have been linked to the different signaling pathways used by IFNs in immature and mature DCs (STAT1 *versus* STAT4, respectively).

### 3.3. Antigen Processing for MHC II-Restricted Presentation

Immature DCs constitutively capture extracellular material by macropinocytosis, phagocytosis and receptor-mediated endocytosis. Extracellular proteins, along with components produced by the DCs themselves, are degraded in endosomal compartments and presented on the cell surface as antigenic peptides bound to MHC II molecules. Upon maturation induction, DCs are very efficient at presenting antigens captured at the time of activation, but their capacity to present subsequently encountered antigens via MHC II is severely reduced, due to inhibition of MHC II synthesis and consequent shutdown of antigen processing function. Simmons and colleagues recently showed that, in contrast to TLR-activated DCs, immature DCs exposed *in vitro* to type I IFNs (Setting 3) acquire a mature phenotype without down-regulating MHC II synthesis and turn-over [61]. In particular, DC maturation induced by IFN-α is characterized by sustained MHC II synthesis and intracellular localization as well as higher MHC II surface levels in parallel with decreased degradation (Table 2). This evidence has been obtained with both murine Flt-3L- and GM-CSF-cultured BM DCs and human GM-CSF-cultured BDCA-1^+^ myeloid DCs induced to mature with type I IFNs, and suggests that these cytokines drive a distinctive DC maturation program allowing continued sampling of antigens for presentation. This may result in increased opportunity to present antigens that are experienced by DCs at different times and locations.

The effect of type I IFNs on MHC II-restricted presentation has also been studied in human DCs generated *in vitro* with IFN-α (Setting 1). As described above, these cells preferentially express LOX-1, which is only barely detectable in IL-4-DCs, and down-regulate its expression upon LPS-induced maturation. IFN-induced LOX-1 was demonstrated to play a crucial role in MHC II-restricted presentation of apoptotic cells and CD4^+^ T cell stimulation [40].

An important functional aspect of type I IFNs, which is different from the well-known function of promoting antigen presentation, is the recent demonstration of IFN-induced immunosuppression [76,77]. Indeed, inhibitory effects on viral antigen presentation and virus-specific CD4^+^ T cell responses have been documented upon prolonged exposure to type I IFNs. Specifically, a reduced antiviral response has been observed in mice persistently infected with lymphocytic choriomeningitis virus (LCMV), and this unresponsiveness has been associated with the IFN-mediated induction of IL-10 and programmed cell death 1 ligand 1 (PD-L1) expression in DCs [76,77]. A complete recovery of the antiviral and antitumor properties of type I IFNs was reported after blocking of IFN signaling or PD-L1 expression both in LCMV-infected mice and in patients under IFN-β therapy [76,77,78]. Taken together, these observations indicate that type I IFN-induced immunosuppression is associated with high serum levels and sustained IFN signature occurring during persistent virus infections or after prolonged IFN therapy.

### 3.4. IFN-DC-Mediated T Cell Responses

Type I IFNs play an important and multifaceted role in regulating adaptive responses through both direct and indirect, APC-mediated, effects. These cytokines directly enhance the development of CD4^+^ and CD8^+^ T cells with central memory characteristics, while also contributing to effector memory cell development via collaboration with other cytokines or feedback by APCs. Type I IFNs also ensure the proper differentiation of Th1 cells by restricting the development of alternative Th2 and Th17 subsets [79]. Moreover, a large number of studies outlined their role in prolonging survival and conferring a proliferative advantage to viral- and tumor-specific CD8^+^ T cells [8], leading to the recent finding that prolonged surface expression of CD25 is involved in these IFN-mediated effects [80]. In addition to these direct effects, many studies have highlighted the ability of type I IFNs to modulate T cell response and shape adaptive immunity through their action on DCs (Table 1 and Table 2). As discussed above, IFN-DCs exhibit the phenotype of partially mature DCs. Due to these features, IFN-DCs are *per se* capable to prime T cell responses not only *in vitro* but also *in vivo* [21,22,46,47]*.* Additional stimulation with TLR ligands or T cell derived stimuli has been described either to further enhance the allostimulatory capacity of these cells [25] or to be dispensable for optimal APC function, depending on the experimental conditions [35].

IFN-DCs are capable of promoting Th1 type immune responses through the expansion of CD4^+^ and CD8^+^ T cells producing large quantities of IFN-γ. Cytokines belonging to the IL-12 family (IL-23 and IL-27) have been shown to play a role in this Th1 promoting activity [42,81] as they are produced at high levels by IFN-DCs and are important in enhancing IL-12-mediated CD8^+^ T cell response [82], the number of specific IFN-γ-producing CD8^+^ T cells [83] and CTL survival/proliferation [84].Since their first description, IFN-DCs have been assessed for their allostimulatory capacity with respect to IL-4-DCs. Although in earlier studies IFN-DCs proved to stimulate allogeneic T lymphocyte proliferation to a similar degree as IL-4- DCs [22], subsequent studies carried out by other groups, including ours, highlighted a superior capacity of IFN-DCs with respect to IL-4-DCs to stimulate IFN-γ production in DC-T cell co-cultures [21,27]. Subsequent studies revealed additional differences between IFN-DCs and IL-4-DCs in terms of induction of T cell responses. In particular, Carbonneil and colleagues reported that IFN-DCs exhibit a superior capacity to induce allogeneic CD8^+^ T cell proliferation but stimulate less efficiently CD4^+^ T lymphocytes as compared with immature IL-4-DCs [45]. The different behavior of IFN-DCs was at least in part attributed to their capacity to spontaneously produce IL-10. Indeed, neutralization of IL-10 significantly increases the expression of DC-LAMP and of MHC II molecules, as well as the ability to stimulate CD4^+^ T lymphocyte proliferation. IFN-DCs were found to induce higher levels of apoptosis of CD4^+^ T cells, a feature more striking for naive T cells, that may explains their inferior capacity to stimulate CD4^+^ T cell proliferation [45]. As a consequence of the different pattern of cytokine secretion, IFN–DCs induce T cells to produce type 1 (IFN-γ) and type 2 (IL-4 and IL-10) cytokines, while IL-4–DCs induce only Th1 cell differentiation [25]. Similarly, IL-3-IFN-β DCs also induce IL-5 production in mixed leukocyte culture and are more efficient than IL-4- DCs in that respect [33].

IFN-DCs were also reported to induce a differentiation bias of naive CD4^+^ T cells towards Th1 and Tr1 cells that is independent of their capacity to spontaneously produce IL-10 [45]. Studies aimed at characterizing the relationships between IFN-DCs and T regulatory cells highlighted that polyI-C-stimulated IFN-DCs produce high levels of IL-6 with respect to IL-4-DCs which contributes to the IFN-DC stimulated IFN-γ production by T cells. The release of IL-6 by IFN-DCs has been reported to contrast the suppressive effect of regulatory T cells on IFN-γ production [85].

Priming of naive CD4^+^ T cells with autologous IFN-DCs in the presence of either SEA or anti-CD3, resulted, in addition to a prominent expansion of CXCR3^+^ IFN-γ producing CD4^+^ T cells, in the emergence of two distinct subsets of IL-17-producing cells: (i) a predominant Th17 population selectively producing IL-17 and expressing CCR6; (ii) a minor Th1/Th17 population, producing both IL-17 and IFN-γ [44]. After phagocytosis of apoptotic cells, IFN-DCs can induce Th17 cell expansion and IL-17 release. Notably, the use of neutralizing antibodies revealed that IL-23 was an essential cytokine in mediating Th17 cell development by IFN-DCs. The demonstration of the IFN-DC-induced expansion of both Th1 and Th17 cell populations reveals the intrinsic plasticity of these cells in orienting the immune response and provides a mechanistic link between IFN-α and the onset of autoimmune phenomena, which have been correlated with both IL-17 production and exposure to IFN-α [44].

## 4. Type I IFN Signature

Transcriptional and post-transcriptional control of gene expression has been proven to play an indispensable role in immune responses by regulating the development of most immune cells, including APCs and their responses to pathogens [86,87,88,89,90]. MicroRNA (miR), an abundant class of evolutionarily conserved small non-coding RNA, have been recently identified as important players in the post-transcriptional control of gene expression [91] and a growing body of reports highlighted their role in the regulation of DC generation and functions [92,93,94]. In this section we will review recent studies outlining the still poorly explored relationships among type I IFNs, gene and miR expression and DC functions.

### 4.1. Gene Expression Profiles

A number of gene expression studies have recently been performed to detect type I IFN-specific signature and to understand in greater detail the mechanisms of action of type I IFNs in DCs. In some cases, microarray technologies were used to compare gene expression profiles of IFN-DCs (Setting 1) with classical IL-4-DCs or different conditions of differentiation/maturation [26,40,95,96]. The ensemble of these studies significantly incremented the list of genes known to be induced by IFNs, known as IFN-stimulated genes (ISGs) (Table 3). The complex network of ISGs, initially categorized in 2001 [97], is now organized into a comprehensive database, named Interferome [98]. Other solutions such as Innate Immune Database (IIDB) and related resources [99,100] allow researchers to readily query a disease, treatment, or otherwise modified condition of interest against the known immune response.

Although gene expression studies do not provide conclusive information about cell function, they turned out to be pivotal in defining predominant signaling pathways and transcription patterns induced by type I IFNs in DCs. The functional classification of the IFN signature genes detected in these studies strongly suggested that IFN-induced DC maturation is biased toward a Th1-polarized immune response [27,37,81]. Since IFN-dependent signaling is time and cell type dependent [101], it is important to keep in mind again that different methods and/or stimulation of different cell types will yield different results. For instance, it has been reported that type I IFNs induce phosphorylation of STAT1 or STAT4 in immature and mature DCs, respectively (Setting 3) [62]. Furthermore, early studies on gene expression profile in IFN-α exposed DCs revealed the existence of donor-specific qualitative and quantitative differences in ISG expression in response to this cytokine [102]. However, comparative analysis of genes up-regulated in immature *versus* mature DCs exposed to IFN-α, revealed quantitative more than qualitative differences in ISGs, suggesting the existence of a “core” signature of IFNs. In fact, IFN-*α* induces a pattern of expression that is similar to the majority of the ISGs in both immature and mature DCs, but that is different from those of all other methods of maturation [62]. Likewise, Mazous and colleagues analyzed the features of type I IFN-induced DCs generated in the presence of either IL-3 or GM-CSF (Setting 1), and compared their capacity to respond to poly(I:C) and to subsequently trigger T cell activation [31]. Gene expression analysis disclosed that both DC types expressed the same pattern of genes, including cytokines (IL-6, IL-1β, IL-10), chemokines and chemokine-receptor genes (CCL20, CCL3, CCL5, CXCR4, CCR5, CCR2). Genes encoding molecules involved in T cell/DC interactions (CD44), antigen recognition/uptake (TLR2, TLR4, CLECSF12/Dectin-1), processing (PRG1 and TAP1), antigen presentation (β-2 microglobulin, CD74, CD1a, CD68, LAMP-3), signal transduction (NFkB2), protection against oxidative stress (SOD2) and motility (Cdc42, IFIT1) were also transcribed. Conversely, it has been recently reported that monocytes differentiated into DCs using GM-CSF and different type I IFN subtypes (Setting 1) show differential gene expression profiles. Transcriptome analyses identified 78 genes differentially expressed between IFN-α-DCs and IFN-β-DCs [32]. Interestingly, many of the differentially expressed genes are important players in DC biology, including several chemokines, such as CXCL11, some receptors involved in the process of phagocytosis (FCGR, MARCO, CLEC5A), molecules implicated in induction of tolerance (DEFB1, IDO1). However, these differences are unlikely to impact the efficacy of T cell functional response since IFN-α-DCs and IFN-β-DCs were equipotent in inducing the proliferation and polarization of allogeneic naive CD4^+^ T cells into Th1 cells, and in stimulating autologous antigen specific CD4^+^ or CD8^+^ T cells [32].

In recent years, novel and unexpected ISGs, characterized in different experimental settings, were added to the list of possible mediators of type I IFN immunomodulatory activity. In this regard, our group analyzed the specific signature of IFN-DCs to define the molecular mechanisms underlying their distinct functional activity both *in vitro* and *in vivo* [40]. Starting from the results of global microarray gene profiling analysis in IFN-DCs (Setting 1), as compared with IL-4-DCs, these authors discovered that LOX-1 overexpression in IFN-α-DCs correlates with increased levels of genes belonging to immune response families and high competence in inducing T cell immunity against antigens derived from allogeneic apoptotic lymphocytes.

Other genes whose expression has been found to be enhanced in IFN-DCs as compared to IL-4/TNF-α-DCs are genes strongly related to NK cell functions (TRAIL, granzymes, KLRs and other NK cell receptors) [26], together with markers of DC activation and migration to the lymph nodes (DC-LAMP, CCR7 and CD49d) [26]. Interestingly, the higher expression of genes of the IFN pathway (STAT1 and IRF7) as well as high surface levels of CD123 and low levels of the myeloid marker CD209, resemble the expression pattern of pDCs [103,104,105], in line with the results of Mohty and coworkers describing the expression of other pDC markers like TLR7 in IFN-DCs (Setting 1) [27]. Therefore, IFN-DCs have signs of a pronounced maturation and show a more plasmacytoid phenotype associated with NK cell characteristics at molecular and protein level.

In the attempt to understand in greater details the mechanisms of action of type I IFNs *in vivo*, in both clinical and physiopathological settings, several studies outlined a clear IFN-α signature in patients experiencing several autoimmune disorders, including psoriasis, multiple sclerosis (MS), rheumatoid arthritis, dermatomyositis, primary biliary cirrhosis, and insulin-dependent diabetes mellitus [106,107]. Of interest, the comparison of the modulations observed in human peripheral blood mononuclear cells (PBMCs) isolated after the *in vivo* administration of IFN-α with the changes occurring in PBMCs or purified monocytes isolated from healthy donors and exposed *in vitro* to the cytokine revealed a significant correlation among the IFN-α-up-modulated genes in the various groups [108]. Understanding these signatures, the disease-specific differences and the biological effects induced has the potential to facilitate IFN-driven therapeutic development.

Finally, as expected on the basis of its antiviral activity, IFN-α signatures have been identified as prominent aspects of many transcriptional profiles involved in the response to pathogens in primates [109].

### 4.2. microRNA Expression Profiles

As IFN system represents an integral part of the mammalian innate immunity critical in the response to pathogens, it is not surprising to find that cellular, IFN-regulated miRs contribute to this antiviral defense or are involved in the cellular response to these pleiotropic cytokines (Table 3). Recently, IFNs have come into view as strong regulators of miRs contributing to antiviral innate immunity [110]. In particular, it has recently been reported that DC differentiation driven by IFN-α is characterized by a specific miR signature composed of 10 miRs, of which miR-23a, miR-27b, miR-30, miR-32, miR-100, miR-146a and miR-125b exhibit a significant down-modulation, whereas miR-7 and miR-155 are remarkably up-modulated [111]. Importantly, IFN-DCs (Setting 1) and IFN-α-treated peripheral pDCs display the same pattern. Conversely, IL-4-DCs do not exhibit any significant modulation of these miRs, confirming the potent and specific activity of IFN-α to modulate selected miR during DC functional differentiation [112]. Of interest, miR-23a and miR-125b were proven to be negatively associated with up-modulation of Blimp-1 occurring during IFN-α-driven DC differentiation. Moreover, miR-155, one of the miR mostly in use in immune signaling pathways [113], is significantly over-expressed in IFN-DCs, representing a marker of the highly activated phenotype of these cells. Among the other IFN-α-driven miR, miR-146a has been reported to negatively regulate DC cross-priming by suppressing IL-12p70 production and its up-regulation, following oxLDL stimulation, and to inhibit the pro-inﬂammatory cytokine release and maturation of DCs [83,114]. Hence, the marked decrease of miR-146a in IFN-DCs suggests an additional mechanism by which IFN-α promotes DC functional properties [111].

**Table 3:** Differentially expressed genes and miR in type I IFN response.

Biological sample	Stimulus	Differentially Expressed gene	Ref.	Biological sample	Stimulus	Differentially Expressed microRNA	Ref.
IL4/IFN-DCs (Setting 2)	IFN-α	CXCL-9, CXCL-10, CXCL-11, MxA, MxB, ISG-15, ISG-56K, STAT-1, IRF7, PKR, 2-5OAS, IFP35, BST2	[102]	IFN-DCs (Setting 1)	IFN-α	↓ miR-23a; miR-27b; miR-30c; miR-32; miR-100; miR-146a; miR-1 25b; miR-let7e.	[111]
						↑ miR-155; miR-7	
IL4-DCs (Setting 3)	IFN-α/ β TNF α/ PGE_2_/ IFN-α/β	STAT1 STAT4	[62]	pDCs	IFN-α	↑ miR-155 ↓ miR-155 *	[114]
				pDCs	IFN-α/β	↑ miR-146a	[115]
IFN-DCs (Setting 1)	IFN-α/ω IFN-β	CXCL11 FCGR, MARCO, CLEC5A, DEFB1, IDO1	[32]	MDMs	IFN-α/β	↑ miR-28; miR-125b; miR-150; miR-382	[116]
IFN-DCs (Setting 1)	IFN-α	LOX-1	[40]	IL4-DCs (Setting 3)	RSV/ IFN-β *	↑ miR Let7b	[117]
IFN-DCs (Setting 1)	IFN-α	TLR7	[27]	PBMCs	Type I IFNs	↑ miR146a	[118]
IFN-DCs (Setting 1)	IFN-α	TRAIL, granzymes, KLRs and other NK cell receptors, DCLAMP, CCR7 and CD49d	[26]	PBMCs	MS/ IFN-β **	↓ mir-29 family	[119]
IFN-DCs (Setting 1)	IFN-β	IL-6, IL-1β, IL-10, CCL20, CCL3, CCL5, CXCR4, CCR5, CCR2,CD44, TLR2, TLR4, CLECSF12, PRG1, TAP1, β2 microglobulin, CD74, CD1a, CD68 LAMP-3, NFkB2, SOD2, Cdc42, IFIT1	[31]	PBMCs (healthy donors)	IFN-α	↑ miR-1; miR-30; miR-128; miR-196; miR-296;	[120]
		
				PBMCs (CHC)	IFN-α **	↑ miR-1; miR-30; miR-296;	

Notes: A summary of representative type I IFN modulated genes (left column) or miR (right column). For a complete list of data, see References. * viral –induced IFN; ** IFN therapy in clinical settings; RSV, respiratory syncytial virus; MS, multiple sclerosis; SLE, systemic lupus erythematosus; CHC, chronic hepatitis C.

Additional studies showed that type I IFNs are involved, in autocrine/paracrine way, in the inverse regulation of miR-155* and miR-155 expression, as well as of miR-146a, in pDCs [114,115]. The expression of miR-155 is slightly up-regulated, whereas that of miR-155* and pri-miR-155 are down-regulated by stimulation with IFN-α over a 24 h time course. These studies identified and validated novel miR-IFN networks involved in the regulation of pDC activation.

In the context of antiviral response, it has been reported that several IFN-induced miR (miR-1, miR-30, miR-128, miR-196, miR-296) are basally present as well as induced by type I IFNs to varying degrees in PBMCs from healthy individuals and from chronic human HCV-infected patients [120]. Recently, it has been shown that respiratory syncytial virus RSV infection of primary DCs up-regulates cellular miR responses (miR-let7b) through an IFN-dependent mechanism [117]. In addition, the up-regulation of four anti-HIV-1 miR (miR-28, miR-125b, miR-150, miR-382) was reported in macrophages treated with both IFN-α and IFN-β, and was shown to partially contribute to inhibition of HIV-1 infection in these cells [116]. Of note, several IFN-induced miR have been demonstrated to have *per se* direct anti-viral activity. For example, at least 8 miR, expressed in response to IFN-α, display RNA sequence specificity with the HCV genome, and of these, miR-351, miR-431 and miR-488 can functionally inhibit HCV replication [121]. Similarly miR-29a, which is also IFN-α/β inducible, has homology to the HIV genome 3’-UTR [122]. Thus, IFN-inducible miR that interact with HCV and HIV genomes and limit virus replication serve as clear examples that some miR are critical components of the IFN response to infection. Finally, it is important to note that IFNs also directly regulate the expression of the miR biogenesis machinery, since they post-transcriptionally repress Dicer protein production, in contrast to IFN-γ, which instead induces its expression [123].

In the clinical setting, data on type I IFN signature of miR were validated as *in vivo* biomarkers in the context of some diseases, such as MS or systemic lupus erythematosus (SLE). In a recent study, by Hecker and colleagues, microarrays were used to investigate the expression dynamics of miR in PBMCs of patients with MS in response to IFN-β therapy [119]. They observed that the up-regulation of IFN-β-responsive genes is accompanied by a down-regulation of several miR, including members of the miR-29 family. These differentially expressed miR were found to be associated with apoptotic processes and IFN feedback loops [119]. Similarly, by comparing the miR profile in PBMCs from patients and control individuals, a SLE-specific signature associated with disease activity has been observed. Most differentially expressed miR were predicted to target genes that regulate cell proliferation, differentiation and apoptosis and are mediators of the type I IFN pathway [118].

Finally, more recent progress in the biology of the IFN system points to epigenetic modulations as another component of IFN-induced innate immune response, suggesting the existence of a not yet fully explored “epigenetic signature” in genes activated by IFNs [124,125,126].

## 5. Conclusions

Type I IFNs have a long record of clinical use and are still currently utilized in medical practice in patients with certain types of cancer, viral infections and chronic inflammatory diseases. For a long time, the importance of the effects of type I IFNs on cells of the immune system was neglected. An ensemble of data stemming from early and recent studies conducted in both mouse and human models now indicate that these cytokines can act as effective vaccine adjuvants in the induction of protective immunity by virtue of their “natural alliance” with DCs [127]. In fact, DCs have been identified as the key cellular targets of type I IFNs in the development of protective responses to immunogenic tumors [128] as well as in the priming of humoral immunity and induction of long-term immunological memory [68]. Likewise, these cytokines turned out to be critical mediators in the spontaneous priming of antitumor CD8^+^ T cell responses *in vivo* [129]. General consent has then been achieved on the ability of type I IFNs to promote DC generation and functional activation both *in vitro* and *in vivo*. However, if we consider the overall *in vitro* data on how type IFNs can affect DC biology, some apparently contrasting results have been reported due to the different activity that these cytokines may exert on cells at different stages of differentiation (monocytes *versus* immature DCs), and to their interaction with other immune mediators. Likewise, *in vivo* studies unraveled that type I IFNs can exhibit beneficial effects, including antitumor and antiviral activities, but also adverse effects leading to auto-reactive CD8^+^ T cell responses or immune suppression by inhibiting virus-specific CD4^+^ T cell responses. Notably, this peculiar type of IFN-induced inhibitory effect, which may apparently be in contrast with the general role of type I IFNs as enhancers of protective immune responses, is associated with high serum levels and sustained IFN signature occurring during persistent virus infections or after prolonged IFN therapy [76,77,78].

**Figure 2:**
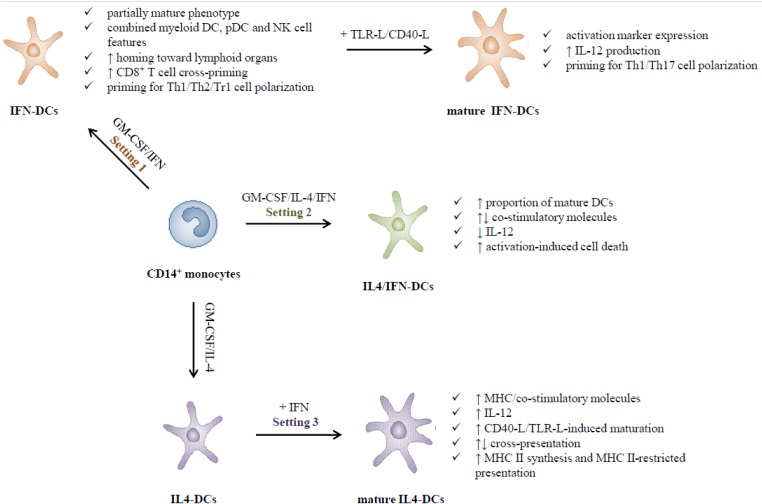
Schematic model of functional outcomes of type I IFN-APC interaction.

In this review, we discussed data on the interactions of human DCs with type I IFNs in different experimental settings, highlighting the functional outcomes and outlining the role of these cytokines as potent regulators of DC differentiation and functional activities and of their capacity to shape adaptive immunity, as schematically depicted in Figure 2. The type of immune response elicited by DCs can be manipulated during their *in vitro* generation by modulating culture microenvironment, antigen type and delivery route as well as maturation stimuli. Special attention should also be given to the modulation of type I IFN receptors occurring during the DC differentiation/maturation process [50]. It should be emphasized that the prompt production of type I IFNs in response to pathogen attack and tumor-related signals points to these cytokines as the critical early players in the differentiation and activation of DCs not only in *in vitro* settings but also in *in vivo* conditions. The knowledge that monocyte-derived type I IFN-conditioned DCs show distinctive traits, associated with the induction of a potent Th1 polarized immune response, CD8^+^ T cell cross-priming and peculiar transcriptional signatures, suggests that they may resemble natural DCs rapidly occurring *in vivo* in response to danger signals. Furthermore, the results achieved on type I IFN-DC interactions support the concept that type I IFNs represent a powerful natural adjuvant for the connection between innate and adaptive immunity, by acting on DC differentiation/activation [130]. Therefore, *in vitro* generated DCs can represent an attractive new tool for the development of more effective immunotherapeutic strategies in patients with cancer and some chronic infectious diseases. However, the current knowledge on the different aspects of IFN-DC interactions reveals the Janus face of the IFN system, highlighting the need of further basic and clinical research in order to fully exploit the therapeutic potential of this complex natural alliance.
